# 

**Published:** 1917-04-07

**Authors:** 


### MULTIPLE STETHOSCOPES.

Safeguarding ourselves with a remark as to the much greater frequency of alleged than real novelty in the matter of surgical instruments, we noticed in the last number but one of The Hospital a contrivance to which the name "Stethodyoscope" had been given, its object being to allow a student to auscult simultaneously with his teacher. Dr. W. H. Coupland, of Lancaster, who has published several papers dealing with historical medical subjects, is now kind enough to furnish a reference to a work where are figured no less than twelve students using together one stethoscope. The saving in time and, we may add, in wear and tear of the patient thus obtained will be obvious.

### HOSPITAL AND INSTITUTIONAL RESULTS.

**Wolverhampton and Staffordshire General Hospital.**—While the daily average number of occupied beds has remained about stationary, the average cost has risen from £64 in 1914 to £82 in 1916. The income last year was £19,910, and the expenditure, £16,903, both amounts being the largest in the history of the hospital.

**Crichton Royal Institution, Dumfries.**—There were 935 patients in the institution at the close of the year; the recovery rate for the year was 62.1 per cent. The physician-superintendent's report contains a description of the weather throughout the year; it rained on 232 days, but the sun shone on 280 days. The list of members of the staff who have joined the Colours occupies six pages of the report.

**Blackburn and East Lancashire Royal Infirmary** —The infirmary commenced the year with an adverse balance of £3,097, which during 1916 was increased to £3,378. The average cost per occupied bed has increased from £57 12s. 7d. in 1910 to £73 0s. 3d. in 1916. The report is printed in full, and contains a portrait of Mr. R. A. Yerburgh, the President, who died on December 18 last.

**City of London Lying-in Hospital.**—The report has been considerably abbreviated for the sake of economy. 1,190 women were delivered in 1916, being 179 more than in the previous year; 22 women had twins. Total income, £6,938; total expenditure, £7,612. One legacy only, £50, was received.

**Royal Edinburgh Mental Hospital, Morningside** One thousand three hundred and thirteen patients were under treatment during the year, 922 remaining on December 31. In the experience of the physician-superintendent there has been no increase of insanity in the civil population, although it was expected to see more cases alleged to be due to bereavement, the result of the war. The Zeppelin raid was responsible for the admission of three cases.

**Royal Hospital for Diseases of the Chest, City Road, E.C.**—The in-patient department of the hospital during the war is an auxiliary of the City of London Military Hospital, Homerton. The out-patient department continues on usual lines; the attendances have fallen from 17,774 in 1915 to 15,987 in 1916. The total income, £10,079, included £3,467 payments by the War Office, and exceeded the expenditure by £1,072.

**Queen Charlotte's Hospital.**—Good progress is reported in the work of the Ante-Natal Department, and additional accommodation has been provided. It has now been decided to establish an Infant Consultation Centre. Sixty-one medical students entered the Midwifery Training School as compared with fifty-three in 1915. Entries for training as pupil midwives and monthly nurses were much lower than in the previous year. Total income, £9,308; total expenditure, £8,284.

**Birmingham and Midland Eye Hospital.**—At the recent annual meeting Mr. Neville Chamberlain, the Director of National Service, was re-elected President. The annual report was presented and the accounts showed an excess of ordinary expenditure over ordinary income of £501. Legacies amounting to £1,049 were received in 1916. Of 20,990 out-patients, 17,689 were insured persons.

**General Hospital, Tunbridge Wells.**—The average weekly treatments in the electrical *x*-ray department were 210. The income from all sources amounted to £7,639, and the total expenditure was £8,160. A legacy of £900 and a donation of £500 for endowment purposes have been invested.

### GIFTS AND BEQUESTS.

Mr. F. W. Barrows, J.P., of Leamington, left a number of legacies to missionary and other societies, and the residue of his estate to the Birmingham Hospital Sunday Fund.

Mr. W. O. Marriott, of Nottingham, subject to some small personal legacies, left estate valued at £8,085 gross. Two-tenths of the estate were left to the General Hospital, two-tenths to the Children's Hospital, one-tenth to the Women's Hospital, all at Nottingham; one-tenth each to Guy's and St. Thomas's Hospitals, London; and the remainder to other charities.

Mr. John Holmes, of Bradford, left £500 to Bradford Royal Infirmary.

Sir George Franklin, of Sheffield, left £5,000 to the board of management of the Sheffield Queen Victoria Nursing Association.

The Warneford and South Warwickshire General Hospital has been left £2,000, duty free, by Mr. B. W. Jones, of "Brockhurst," £1,000 of which is to endow a bed in his memory, and £50 by the Rev. J. L. Clarke. Mr. R. W. Emmons, of Leamington Spa, has given the hospital £100 by request of his late wife.

Miss Mary Martha Hirst, of Wood Cottage, Meltham Mills, Huddersfield, left £500 to the Meltham Mills Convalescent Home and £50 to the Institute for Trained Nurses for the Town and County of Leicester.

The Chelsea Hospital for Women has received for its Rebuilding Fund a donation of £105 from the Trust Fund of the late E. Schumacher, Esq.

The Bradford Dyers' Association, Ltd., at a recent meeting, gave a large number of donations, ranging from £5 to £125, to hospitals and other charities, chiefly in Yorkshire and Lancashire.

Mr. Edward Hinkley, of Southport, has left, amongst a large number of legacies, £1,000 each to the Southport and Birkdale District Nursing Society, the Leicester Infirmary, Dr. Barnardo's Homes, the Homœopathic Dispensary, Southport, and the Lifeboat Institution.

Mr. Edward H. Langdon has endowed a cot at the Manchester Northern Hospital for Women and Children, in memory of his son, Captain Wilfred Langdon, who was killed in action in France.

Lord Fitzhardinge, who died on December 5 last, left £1,000 to the Berkeley Cottage Hospital.

Mr. William Petersen has given £1,000 to All Saints' Hospital for Genito-Urinary Diseases to endow a bed in memory of his son, who was killed in action.

Mrs. Elizabeth Hewetson has left, subject to the life interest of a sister, £333 6s. 8d. each to the Lifeboat Institution, the British Home for Incurables at Streatham, and the National Hospital for the Paralysed and Epileptic, and £166 13s. 4d. to fourteen other charities.

The late Mayor of Sunderland (Alderman Stansfield Richardson) bequeathed £500 to the Sunderland Royal Infirmary and several other legacies to Sunderland charities, including £100 to the Sunderland District Nursing Association.

Mr. H. Osborne O'Hagan, of Hampton Court, has given 200 guineas to Hampton Court Auxiliary Military Hospital.

Mr. Thomas Evans, of Birmingham, whose will has just been proved at £28,861, left nearly all his property to the Birmingham Corporation, the income each year to be distributed between the hospitals and charitable institutions of the city by the Lord Mayor

The late Mr. Benjamin Turner, an architect and surveyor of Barnsley, has left £100 each to the Beckett Hospital and the Leeds General Infirmary.

A new ward has been provided at a cost of £1,000 at the Huddersfield War Hospital by Mr. F. W. Sykes.

Mr. William Wright, of Hove, has left £500 to Dr. Barnardo's Homes, £400 to the Royal Victoria Institution in Newcastle for the Industrious Blind, and £250 to the Ingham Infirmary.

Mrs. Mary Barnes, of Wilmslow, left £500 each to the Royal Infirmary, the Lunatic Hospital, and the Dispensary at Manchester, the Children's Hospital at Pendlebury, the Ancoats Hospital, and the Ardwick and Ancoats Dispensary. These institutions will also receive part of the residue, subject to a life interest.

Queen Mary's Convalescent Auxiliary Hospitals, Roehampton, have received £1,000 from the Saskatchewan Branch of the Canadian Red Cross to maintain a ward for a year.

Mrs. Catherine Lowden, of Harrogate, left £1,000 to the Leeds General Infirmary, and £500 each to the Hospital for Incurables at Manchester and the Cookridge Convalescent Home.

Mrs. Emma Georgiana Hill, of Carmarthen, who died on January 16 last, left £200 to the Carmarthenshire Infirmary.

Mr. Joseph Giles, of Tilehurst, Berks, left £1,000 each to the Cancer Hospital, Fulham, and the Royal Berks Hospital, Reading.

Mr. C. J. Gwyn, a wine and spirit merchant, of Ramsgate, left £1,000 to the Ramsgate General Hospital and Seamen's Infirmary.

St. Bartholomew's Hospital has received £1,707, the residue of the estate of the late Mr. W. E. Wright, and £4,000, balance of £10,000 left by Mr. H. L. Florence.

Mrs. Jane Gerlack, who died at Zurich on Sept. 30, has left £1,000 each to the Cancer Hospital and the Chelsea Hospital for Women, and £500 to Brompton Consumption Hospital.

Miss Sarah Ellen Owen, of Maesyrhedydd, Aberystwyth, who died on May 6, 1916, left out of the residue of her estate £500 to Aberystwyth Infirmary and £100 to the Aberystwyth Nursing Society.

---

Messrs. Cassell and Co. are shortly publishing the fourth edition of Mr. Comyns Berkeley's "Handbook on Midwifery." It has been considerably expanded to meet the requirements of the Central Midwives Board, and to render it suitable for obstetric dressers.

Rates of Subscription: Twelve months, 6/6; Six months, 4/-; Three months, 2/-; post free to any address in the United Kingdom. Abroad, Twelve months, 8/8; Six months, 5/-; Three months, 2/6, post free. The Hospital "Index" to Volume LX., April to September 1916, is now ready, price 2½d. per copy, post free. Address The Manager, "The Hospital," The Hospital Building, 28/29 Southampton Street, Strand, London, W.C. 2.

